# Circulating and tumor-infiltrating mucosal associated invariant T (MAIT) cells in colorectal cancer patients

**DOI:** 10.1038/srep20358

**Published:** 2016-02-03

**Authors:** Limian Ling, Yuyang Lin, Wenwen Zheng, Sen Hong, Xiuqi Tang, Pingwei Zhao, Ming Li, Jingsong Ni, Chenguang Li, Lei Wang, Yanfang Jiang

**Affiliations:** 1 Department of Colorectal & Anal Surgery, Changchun, 130032, China; 2 Key Laboratory of Zoonosis Research, Ministry of Education; the First Hospital, Jilin University, Changchun, 130032, China; 3 Jiangsu Co-innovation Center for Prevention and Control of Important Animal Infectious Diseases and Zoonoses, Yangzhou 225009, China

## Abstract

Mucosal associated invariant T (MAIT) cells are important for immune defense against infectious pathogens and regulate the pathogenesis of various inflammatory diseases. However, their roles in the development of colorectal cancer (CRC) are still unclear. This study examined the phenotype, distribution, clinical relevance and potential function of MAIT cells in CRC patients. We found that the percentages of circulating memory CD8^+^ MAIT cells were significantly reduced while tumor infiltrating MAIT cells were increased, especially in patients with advanced CRC. The serum CEA levels were positively correlated with the percentages of tumor infiltrating MAIT cells in CRC patients, but negatively correlated with the percentages of circulating MAIT in advanced CRC patients. Activated circulating MAIT cells from CRC patients produced lower IFN-γ, but higher IL-17. Furthermore, higher levels of Vα7.2-Jα33, IFN-γ and IL-17A were expressed in the CRC tissues. Co-culture of activated MAIT cells with HCT116 cells enhanced IL-17 expression and induced HCT116 cell cycle arrest at G2/M phase in a contact- and dose-dependent manner, which was abrogated by treatment with anti-MR1. Therefore, MAIT cells preferably infiltrate into the solid tumor in CRC patients and may participate in the immune surveillance of CRC.

Mucosal associated invariant T (MAIT) cells are innate-like T cells expressing a semi-invariant T cell receptor (TCR) of Vα7.2-Jα33 chain and a limited array of Vβ2 or Vβ13 chain in humans^1^. Circulating and tissue-infiltrating MAIT cells can be characterized by expressing Vα 7.2 segment with either CD161 or IL-18Rα on cell surface^2^,^3^. MAIT cells are preferably resident in the intestinal mucosa and liver in humans^4^,^5^,^6^. In contrast to conventional T cells that recognize specific antigen peptides, MAIT cells can recognize and respond to microbial vitamin B metabolites in the major histocompatibility complex class I (MHC I)-related molecule (MR1) restricted manner^7^,^8^,^9^,^10^. Co-cultured with bacterium-infected antigen presenting cells (APC), activated MAIT cells can produce diverse cytokines, including interferon-γ (IFN-γ), tumor necrosis factor-α (TNF-α) and interleukin-17A (IL-17A)^3^,^11^,^12^.

It was considered that MAIT cells acquired memory phenotypes after birth and accumulated in the lamina propria of intestinal mucosa in a manner depending on B cells and the commensal flora^13^. However, a recent study in the human fetus indicates that MAIT cells can acquire memory phenotypes before birth, independent of established commensal flora^14^. Previous studies have shown the importance of MAIT cells in host defense against various infectious pathogens^15^,^16^,^17^,^18^. Notably, accumulative MAIT cells protect from TNBS-induced colitis in rodents^19^ and inflammatory bowel disease in humans^20^. Therefore, MAIT cells in the intestinal lamina propria may be natural protectors from infection and inflammation.

Colorectal cancer (CRC) is one of the most common malignant tumors worldwide. The pathogenesis of CRC is attributed to epithelial genetic mutations, impaired mucosal integrity, disordered microbiota and inflammation^21^. CRC usually disrupts the mucosal homeostasis and barrier function. Its development and progression depend on the interaction of neoplasms, pathogens and tumor-infiltrating lymphocytes (TIL) in the tumor microenvironment^22^,^23^. TIL are believed to affect clinical outcome and survival of CRC patients^24^. The intestinal inflammation induced by TIL targeting either CRC or microbia can alter the prognosis of tumor and the microbia compositon^25^,^26^. Previous studies have shown the relationship between different types of natural killer T (NKT) cells and the progression of CRC^27^,^28^. However, the roles of circulating and tumor-infiltrating MAIT cells in human CRC are still unclear.

In this study, we examined the phenotype, distribution, clinical relevance and biological function of MAIT cells in CRC patients. Our findings indicate that MAIT cells preferably accumulate in the solid tumor and are associated with the immune surveillance of CRC.

## Results

### Characterization of circulating MAIT cells in CRC patients

A total of 48 newly diagnosed CRC patients and 22 gender- and age-matched healthy controls (HC) were enrolled in the First Hospital of Jilin University, Changchun, China from August 2013 to September 2014. The demographic and clinical characteristics of 48 CRC patients and 22 HC are summarized in Supplementary Table 1. There was no significant difference in the distribution of age, gender, BMI among these groups of subjects and no significant difference in the tumor location (Colon/Rectum), WBC and lymphocytes between these two groups of CRC patients. The patients with advanced CRC had significantly higher levels of serum carcinoembryonic antigen (CEA) than early stage group.

**Figure 1:**
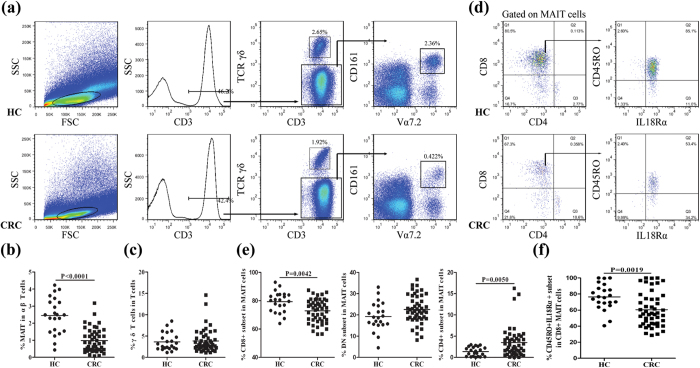
Characterization of circulating MAIT cells. PBMC were isolated from 22 healthy controls (HC) and 48 CRC patients and stained with fluorescent antibodies against CD3, CD161, TCRγδ, and TCRVα7.2. The frequency of CD3^+^TCRγδ^−^Vα7.2^+^CD161^+^ MAIT cells was characterized by flow cytometry. The cells were first gated on living lymphocytes and then on CD3^+^TCRγδ^−^ T cells. The percentages of CD3^+^TCRγδ^−^Vα7.2^+^CD161^+^ MAIT cells in αβ T cells were determined. The different subsets of circulating MAIT cells were gated on CD3^+^TCRγδ^−^Vα7.2^+^CD161^+^ cells and the percentages of CD4^+^ CD8^+^ or CD4^−^CD8^−^ (DN) MAIT cells were determined. Subsequently, the percentages of CD45RO^+^IL-18Rα^+^ MAIT cells in total CD8^+^ MAIT cells was further analyzed. Data are representative charts or expressed as the mean values of individual subjects. (**a**) Flow cytometry analysis of MAIT. (**b**) The percentages of circulating MAIT cells in CD3^+^TCRγδ^−^ T cells. (**c**) The percentages of circulating γδ T cells. (**d**) Flow cytometry analysis of the different subsets of MAIT cells. (**e**) The percentages of CD4^+^, CD8^+^ or DN MAIT cells. (**f**) The percentages of CD45RO^+^IL-18Rα^+^ MAIT cells.

Previous studies have suggested that CD3^+^TCRγδ^−^Vα7.2^+^CD161^+^ T cells can be considered as MAIT cells^29^,^30^,^31^. Accordingly, the frequency of circulating CD3^+^TCRγδ^−^Vα7.2^+^CD161^+^ cells in total CD3^+^TCRγδ^−^ lymphocytes in individual subjects was determined by flow cytometry (Fig. 1a). The percentages of circulating MAIT cells were significantly lower in CRC patients (0.98%, P < 0.0001) than HC (2.45%, Fig. 1b). In contrast, there was no significant difference in the percentages of circulating γδ T cells in CD3^+^ T cells (Fig. 1c). The percentages of circulating conventional CD4^+^ and CD8^+^ T cells in CD3^+^ T cells were similar between the HC and CRC patients (Supplementary Fig. a). Therefore, CRC patients had significantly reduced numbers of circulating MAIT cells.

### Phenotype of circulating MAIT cells in CRC patients

MAIT cells can be subdivided into CD4^+^, CD8^+^ and double negative (DN) subsets^32^. Analysis of circulating MAIT cells in those 48 CRC patients and 22 HC indicated that most of MAIT cells were either CD8^+^ or DN phenotype, and there was a very small proportion of CD4^+^ (Fig. 1d). The percentages of CD8^+^ MAIT cells in total MAIT cells were significantly lower in the CRC patients (72.6%, P = 0.0042) than HC (79.4%, Fig. 1e). The percentages of CD4^+^ MAIT cells, but not DN, were significantly higher in the CRC patients than HC (p = 0.005). Further analysis revealed that the percentages of CD45RO^+^IL-18Rα^+^CD8^+^ memory MAIT cells in total MAIT cells were significantly lower in the CRC patients (60.5%, P = 0.0019) than HC (76.5%, Fig. 1f). Hence, CRC patients had significantly reduced numbers of circulating CD8^+^ MAIT cells, particularly for CD45RO^+^IL-18Rα^+^CD8^+^ memory MAIT cells.

### MAIT cells accumulate in colorectal neoplasms

**Figure 2:**
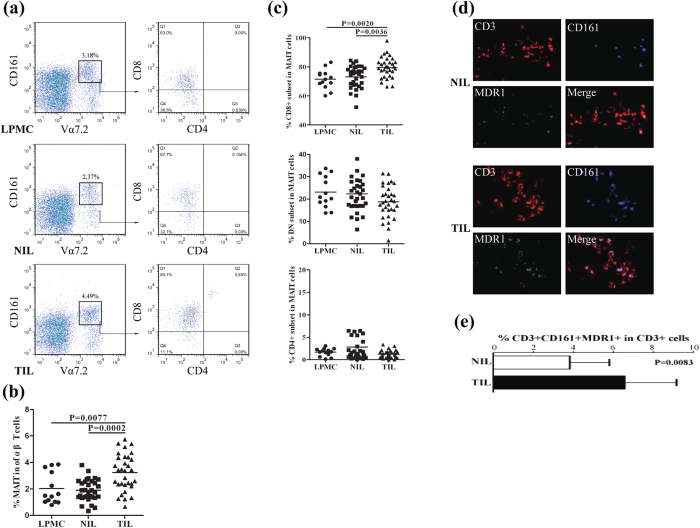
Accumulation of MAIT cells in colorectal neoplasms. A total of 32 freshly surgical CRC tumor tissues were dissected to separat the central neoplasms from the surrounding non-tumor tissues. The tumor-infiltrating lymphocytes (TIL) and non-tumor infiltrating lymphocytes (NIL) were isolated from individual surgical tissues. In addition, 13 lamina propria tissues were obtained from non-tumor patients and their lamina propria mononuclear cells (LPMC) were isolated. Subsequently, the frequency of different subsets of MAIT cells in LPMC, NIL and TIL was determined by flow cytometry. Moreover, the numbers of CD3^+^CD161^+^MDR1^+^ MAIT cells in the central tumors and surrounding non-tumor tissues were determined by fluorescent assays. Data are representative charts, images or expressed as the mean ± SD of individual groups as well as the mean values of individual subjects. (**a**) Flow cytometry analysis. (**b**) The percentages of MAIT cells. (**c**) The frequency of different subsets of MAIT cells. (**d**) Immunofluorescent analysis of MAIT cells in the tumor tissues. (**e**) Quantitative analysis of the frequency of CD3^+^CD161^+^MDR1^+^ cells in total CD3^+^ cells in tissue sections.

The percentages of MAIT cells in lamina propria mononuclear cells (LPMCs), non-tumor infiltrating lymphocytes (NIL) and TIL in the 32 CRC and 16 non-tumor (NT) patients were analyzed by flow cytometry (Fig. 2a). The NT control samples were obtained from another 13 patients with hemorrhoids when they underwent a procedure for treatment of prolapse and hemorrhoids. The demographic and clinical characteristics of those patients are summarized in Supplementary Table 1. There was no significant difference in the values of tumor-unrelated measures between the CRC and NT patients, but there was a significant difference in the tumor-node-metastasis (TNM) stage and serum CEA levels between the early and advanced stages of patients. Furthermore, no significant difference were detected for the percentages of total MAIT cells or CD8^+^ MAIT cells in the LPMC and NIL (Fig. 2b,c). In contrast, the percentages of MAIT cells in CD3^+^TCRγδ^−^ T cells were significantly higher in TIL (3.24%, P = 0.0002 or P = 0.0077) than LPMC (2.03%) and NIL (1.90%, Fig. 2b). In addition, CD8^+^ MAIT cells in the TIL were significantly higher (79.6%, P = 0.0036 or P = 0.0020) than in the NIL (73.1%) or control (71.5%, Fig. 2c).

Next, we determine the distribution of MAIT cells in CRC patients by flow cytometry and tissue staining assays. Due to technical limitations in staining TCRVα7.2 and IL-18Rα in the formalin-fixed paraffin-embedded tissue sections, CD3^+^CD161^+^MDR1^+^ cells were considered as MAIT cells, although they may contain other types of cells. The results showed that the frequency of CD3^+^CD161^+^MDR1^+^ cells in CD3^+^ cells in the tumor sections were significantly greater than that in the non-tumor tissue (P = 0.0083, Fig. 2d,e). The percentages of memory MAIT cells in TIL, LPMC and NIL had no significant difference (Supplementary Fig. b). Previously studies have shown that MAIT cells preferentially accumulate in inflamed mucosa, kidney and brain tumor tissues^20^,^23^,^33^. Because chemokine receptors CCR6 is crucial for T cell trafficking from blood to tissues, especially to the intestine or tumor tissues^12^, Another ten non-tumor controls and ten CRC patients were recruited to stain the frequency of circulating and tissue-infiltrating CCR6^+^ MAIT cells by flow cytometry. The results indicated that high frequency of CCR6^+^ MAIT cells was detected in the controls and CRC patients and there was no significant difference in the percentages of CCR6^+^ MAIT cells between the controls and CRC patients (Supplementary Fig. c). Therefore, an increased number of MAIT cells in the CRC may reflect the migration of circulating MAIT cells into the CRC tissues in CRC patients.

### Stratification analyses of the frequency of MAIT cells in CRC patients

**Figure 3:**
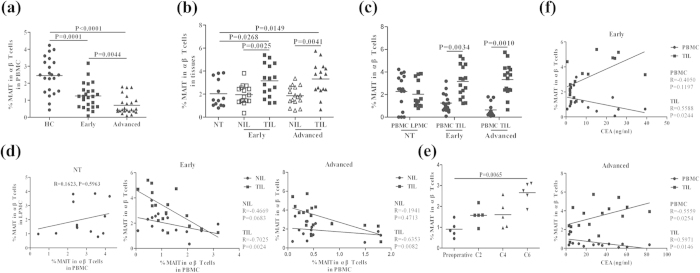
Stratification analysis of the percentages of MAIT cells. The CRC patients were stratified as early (I/II, n = 24) or advanced stage (III/IV, n = 24) and the percentages of MAIT cells in PBMC, NIL and TIL were compared with that in PBMC from 22 healthy controls (HC) and in LPMC from 13 non-tumor patients. Furthermore, the relationship among the percentages of MAIT cells in PBMC, NIL and TIL and the potential association between the percentages of MAIT cells in PBMC or TIL with the levels of serum CEA in individual groups of patients were analyzed. Finally, the percentages of circulating MAIT cells in five advanced CRC patients were characterized longitudinally by flow cytometry following different cycles of chemotherapies. Data are the mean values of individual subjects. (**a**) The frequencies of circulating MAIT cells. (**b**) The frequencies of MAIT cells in LPMC, NIL and TIL from early (n = 16) and advanced (n = 16) stage of CRC patients. (**c**) The percentages of circulating and tissue MAIT cells. (**d**) The relationship between the percentages of circulating and tissue MAIT cells. (**e**) The frequency of MAIT cells following chemotherapies. (**f**) The correlation between the levels of serum CEA and the percentages of MAIT cells in PBMC or TIL in the CRC patients.

Stratification analysis of 48 CRC patients indicated that the percentages of circulating MAIT cells were significantly lower in early stage (1.25%, P = 0.0001) and advanced stage CRC patients (0.71%, P < 0.0001) than HC (2.45%, Fig. 3a). The percentages of circulating MAIT cells in early stage of CRC patients were significantly higher than that in patients with advanced CRC (P = 0.0044). The percentages of MAIT cells in CD3^+^TCRγδ^−^ lymphocytes in TIL from the patients with early (3.16%, P = 0.0025 or P = 0.0268) or advanced stage (3.32%, P = 0.0041 or P = 0.0149) were significantly higher than that in the NIL and LPMC, while there was no significant difference in the percentages of αβ MAIT cells between the LPMC and NIL (Fig. 3b). A similar pattern of the absolute values of MAIT cells was detected in different groups of subjects (Supplementary Fig. d). In addition, there was a significant difference in the percentages of MAIT cells between the PBMC and TIL in CRC patients, but no such difference in NT patients (Fig. 3c). Further analyses revealed that the percentages of circulating MAIT cells were inversely correlated with that in the TIL, (early stage: R = −0.7025, P = 0.0024; advanced stage: R = −0.6353, P = 0.0082), but not in the NIL (Fig. 3d). However, there was no significant correlation of the percentages of MAIT cells between PBMC and LPMC in NT patients. Similar percentages of circulating or tissue MAIT cells were detected in the controls and different stage CRC patients (Supplementary Fig. e).

Five patients with advanced stage of CRC were also recruited in this study, and they had received postoperative chemotherapy with FOLFOX4. The percentages of circulating MAIT cells were characterized longitudinally following chemotherapy. The percentages of circulating αβ MAIT cells gradually increased following the extension of chemotherapy cycles. After six cycles of chemotherapies, the percentages of MAIT cells were nearly normal and were significantly higher than that at pre-operative stage (P = 0.0065, Fig. 3e).

Serum CEA level remains a valuable biomarker for evaluating CRC progression^34^. We examined the potential association between the percentages of MAIT cells and the levels of serum CEA in CRC patients. We found that the percentages of MAIT cells in the TIL were positively correlated with serum CEA levels (advanced stage: R = 0.5971, P = 0.0146; early stage: R = 0.5588, P = 0.0244). In contrast, a moderate negative correlation was found between the percentages of circulating MAIT cells and the levels of serum CEA in the patients with advanced stage of CRC (R = −0.5559, P = 0.0245, Fig. 3f), but not in those with early stage of CRC (R = −0.4050, P = 0.1197).

### Alternation in the levels of Th1/Th17 cytokine secreted by MAIT cells from CRC patients

**Figure 4:**
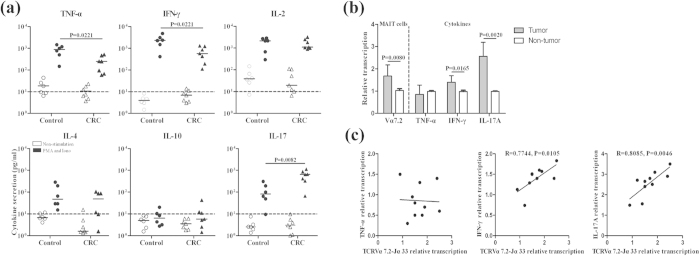
The cytokine profile of MAIT cells in CRC patients. CD3^+^CD161^+^Vα7.2^+^ MAIT cells were purified from six healthy controls or CRC patients and stimulated with, or without, PMA/ionomycin for 48 h. The levels of TNF-α, IFN-γ, IL-2, IL-4, IL-10, and IL-17 in the supernatants of cultured cells were determined by CBA. In addition, the relative levels of Vα7.2-Jα33, TNF-α, IFN-γ and IL-17A mRNA transcripts to the control GAPDH in 10 paired central neoplasm and surround non-tumor tissues were determined by quantitative RT-PCR. The relationship between the levels of Vα7.2-Jα33 and cytokine mRNA transcripts was further analyzed. Data are the mean values of individual subjects or expressed as the mean ± SD of individual groups. (**a**) The levels of cytokines. The dashy lines indicate the detection limits for these cytokines by the CBA. (**b**) Quantitative real-time PCR analysis of the relative levels of Vα7.2-Jα33, TNF-α, IFN-γ and IL-17A mRNA transcripts in the tumor tissues. (**c**) The correlation analysis of the relative levels of Vα7.2-Jα33, TNF-α, IFN-γ or IL-17A in the colorectal neoplasms.

To characterize the function of circulating MAIT cells, MAIT cells were purified from six CRC patients and six age- and gender-matched HC, and stimulated with or without, PMA/ionomycin for 48 h. Subsequently, the levels of TNF-α, IFN-γ, IL-2, IL-4, IL-10 and IL-17A in the supernatants were determined by cytometric bead array (CBA). The gating strategy of flow cytometry for sorting MAIT cells and their cytokine assessment are shown in Supplementary Fig. f. There was no significant difference in the levels of cytokines tested in the supernatants of unstimulated MAIT cells between the patients and HC. However, the levels of TNF-α, IFN-γ, IL-2 and IL-17A in the supernatants of cultured activated MAIT from the patients and controls were significantly higher than that in the unstimulated MAIT cells (Fig. 4a). In comparison with HC, significantly lower levels of TNF-α and IFN-γ, but higher levels of IL-17A were detected in the supernatants of cultured activated MAIT cells from CRC patients. Together, these data suggest that circulating MAIT cells may have Th1- and Th17-biased function and MAIT cells from CRC patients may preferably have Th17-biased function.

To investigate the potential association between MAIT cells and cytokines in colorectal neoplasms, the relative levels of TCRVα7.2-Jα33, TNF-α, IFN-γ and IL-17A mRNA transcripts in the tumor and non-tumor tissues were determined by quantitative real-time PCR. The levels of TCRVα7.2-Jα33, IFN-γ and IL-17A, but not TNF-α, mRNA transcripts in tumor tissues were significantly higher than non-tumor tissues (Fig. 4b). More importantly, the levels of TCRVα7.2-Jα33 mRNA transcripts were positively correlated with IFN-γ and IL-17A in tumor tissues (Fig. 4c). These findings indicated that Th1- and Th17-like MAIT cells infiltrated in the CRC tissues.

### MAIT cells play a role in immune surveillance of tumor via the contact response

**Figure 5:**
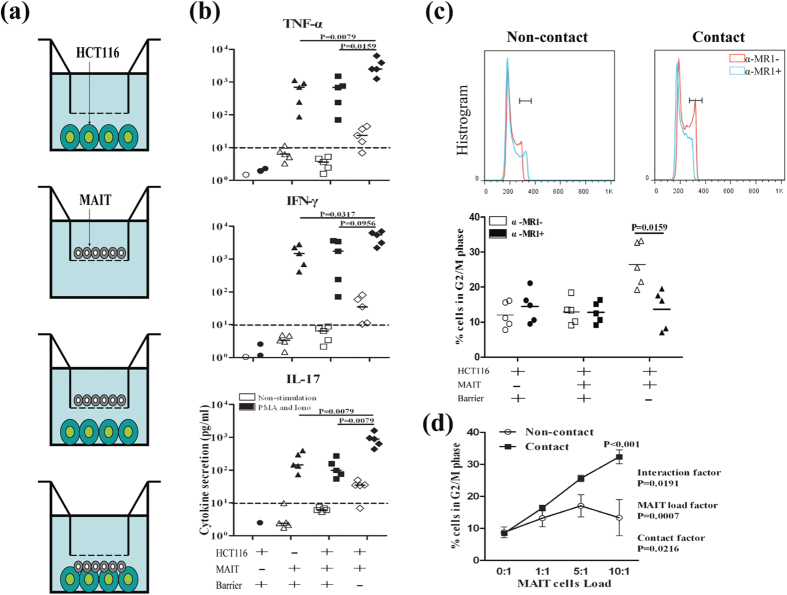
MAIT cells induce the cell cycle arrest of HCT116 in a cell-cell contact-dependent manner. MAIT cells were purified from five healthy controls and stimulated with, or without, PMA/ionomycin for 48 h. The unstimulated or activated MAIT cells were co-cultured with GFP^+^ HCT116 cells in a cell-cell contact or non-contact manner. The MAIT cells or HCT116 cells in medium alone served as the controls, as illustrated in (**a**). Subsequently, the levels of TNF-α, IFN-γ and IL-17 in the supernatants of cultured cells were determined by CBA and the cell cycle status of HCT116 cells was examined by flow cytometry. Data are representative histograms of cell cycling or expressed as the mean values or the mean ± SD of each group from three separate experiments. (**b**) The levels of cytokines. The dashy lines indicate the detection limits for these cytokines by the CBA. (**c**) Analysis of cell cycling in HCT116 cells following co-culture with MAIT cells with or without anti-MR1 (α-MR1). (**d**) Dose curves of MAIT cell activity.

MAIT cells play a role in immune surveillance when activated by bacterium-infected cells and display cytotoxic activity^35^,^36^. The impact of activated MAIT cells on HCT116 cell cycling and proliferation *in vitro* was determined. PBMC were isolated from five healthy donors and MAIT cells were purified by flow cytometry sorting, followed by activated with or without PMA/ionomycin. The activated and unstimulated MAIT cells were co-cultured with GFP^+^ HCT116 cells in 24-well transwell plates at ratios of 10-1:1 for 48 h in a manner of contacting or non-contacting co-culture, respectively. The unstimulated, activated MAIT cells or GFP^+^ HCT116 cells alone served as the controls (Fig. 5a).

Subsequently, TNF-α, IFN-γ and IL-17A levels in the supernatants were determined by CBA and the cell cycle status of HCT116 cells were determined by cell cycling assay. There was no detectable cytokine in the supernatants of co-cultured unstimulated MAIT cells and HCT116 cells regardless of contacting or non-contacting. In contrast, high levels of TNF-α, IFN-γ and IL-17A were detected after co-culture of activated MAIT cells with HCT116 cells either in a contacting or non-contacting manner (Fig. 5b). Interestingly, the levels of IFN-γ and IL-17A in the supernatants of co-cultured activated MAIT and HCT116 cells in a contacting manner were significantly higher than that in other groups.

Analysis of cell cycling revealed that the percentages of HCT116 cells at G2/M phase after co-cultured with activated MAIT cells in a contacting manner were significantly higher than other groups, which was abrogated by treatment with anti-MR1 antibody (Fig. 5c). Subsequently, activated MAIT cells were cultured with HCT116 cells at different ratios in a contacting or non-contacting manner. Following the contacting co-culture, the percentages of HCT116 cells at G2/M phase increased with the increased numbers of activated MAIT cells. Besides, the percentages of HCT116 cells at G2/M phase in the contact group were always higher than that in the non-contact group at the same ratios of MAIT cells to HCT116 cells (P < 0.05 at 5:1; P < 0.01 at 10:1, Fig. 5d). Therefore, activated MAIT induced HCT116 cell cycle arrest at G2/M phase in a contact- and dose-dependent manner.

Further analysis using the EdU incorporation assay indicated that contacting co-culture of activated MAIT cells with HCT116 cells, but not non-contacting co-culture, significantly reduced the viability of HCT116 cells, which was completely abrogated by treatment with anti-MR1 antibody (Supplementary Fig. g). These data indicated that activated MAIT cells significantly reduced HCT116 cell viability in a contact- and MR1-dependent manner *in vitro*.

## Discussion

In this study, we examined the phenotype, distribution and clinical relevance of MAIT cells in CRC patients and explored the potential function of MAIT cells in regulating the cell cycling of human HCT116 cells by co-culture system *in vitro*. Our data indicated that MAIT cells accumulated in the neoplasm and correlated with serum CEA levels in CRC patients. Furthermore, activated MAIT cells produced IFN-γ, TNF-α and IL-17, induced HCT116 cell cycling arrest at G2/M phase and reduced HCT116 cell viability *in vitro*. These findings suggest that activated MAIT cells may participate in the surveillance for CRC. To the best of our knowledge, our findings were the first to describe the characteristics of MAIT cells in the process of CRC.

Characterization of MAIT cells indicated that the percentages of circulating MAIT cells, particularly for CD8^+^ and CD45RO^+^IL-18Rα^+^CD8^+^ MAIT cells in CRC patients were significantly lower than that in HC. In contrast, the percentages of CD8^+^ MAIT cells in TIL, but not NIL, were increased significantly in CRC patients. More importantly, the percentages of circulating MAIT cells were inversely correlated with that in the tumor tissues in CRC patients. These data indicated that circulating activated and memory MAIT cells decreased in CRC patients, but accumulated in the neoplasm, which extended previous observations that a reduction in the frequency of circulating MAIT cells is associated with the infiltration of MAIT cells into the inflammatory and infected tissue^20^,^32^. These data suggest that circulating CD8^+^ MAIT cells may migrate into the tumor tissues during the development of CRC.

To determine the distribution of MAIT cells in CRC patients, we analyzed circulating and tissue-infiltrating MAIT cells by staining with anti-TCR Vα7.2 and anti-CD161 or anti-IL-18Rα^2^,^3^, because CD161 is expressed by the majority of NK cells and diverse subsets of T lymphocytes, including γδ T, most NKT and MAIT cells^37^. However, due to the limitation of available antibodies against TCR Vα7.2, IL18Rα or CCR6 for staining the formalin-fixed paraffin-embedded tissue sections, we stained the tissue sections with anti-MDR1, a well-known biomarker for MAIT cells^16^. Previous studies have demonstrated that MDR1 expression is crucial for the function of MAIT cells^3^,^12^. Accordingly, CD3^+^CD161^+^MDR1^+^ cells were considered as MAIT cells. However, CD3^+^CD161^+^MDR1^+^ cells may include some NKT and NK cells, and the numbers of CD3^+^CD161^+^MDR1^+^ cells we detected may be larger than the counts of absolute MAIT cells. Actually, the percentages of CD3^+^CD161^+^MDR1^+^ cells in total CD3^+^ cells we detected in non-tumor controls (mean 3.8%) were similar to that of CD3^+^Vα7.2^+^IL-18Rα^+^ MAIT cells in total CD3^+^ cells in the intestine^20^. Combining with the data from flow cytometry analysis, our data indicated that MAIT cells accumulated in colorectal neoplasms.

Chemokine receptors of CCR6 and CXCR6 are crucial T cell trafficking from blood to tissues, especially to the intestine or tumor tissues^38^. We analyzed circulating and tissue-infiltrating CCR6^+^ MAIT cells. We found high frequency of CCR6^+^ MAIT cells in both the controls and CRC patients and that there was no significant difference in the percentages of circulating and tissue-infiltrating CCR6^+^ MAIT cells between the controls and CRC patients (Supplementary Fig. c). While CCR6 and CXCR6 expression on conventional CD8^+^ T cells can be up-regulated by ligand engagement^39^, high levels of CCR6 and CXCR6 expression on circulating MAIT cells appear independent of ligand engagement^12^. Because high frequency of MAIT cells expressed CCR6 in non-tumor controls (92.61% in PBMC; 93.11% in LPMC) it was not surprising to observe similar frequency of CCR6+ MAIT cells in TIL (93.49% in NIL; 92.77% in TIL, Supplementary Fig. c). Hence, MAIT cells may persistently express CCR6 and CXCR6 when they migrate into the tumor environment.

We stratified CRC patients and found that the percentages of circulating MAIT cells in patients with advanced CRC were significantly lower than those with early stage of CRC. The percentages of MAIT cells in TIL were significantly higher than that in the NIL in the patients with early or advanced stage of CRC, and they were positively correlated with the levels of serum CEA in patients with advanced CRC, but not in those with early stage of CRC. Furthermore, the percentages of circulating MAIT cells were gradually increased after post-operative chemotherapies. Hence, the decreased frequency of circulating MAIT cells and increased MAIT cells in the tumor tissues may be potential biomarkers for evaluating the severity and prognosis of CRC. Given that the percentages of activated and memory CD8^+^ cells, particularly for IFN-γ-secreting CD8^+^ cells in the tumor tissues are associated positively with the survival of patients with CRC, but negatively with the metastasis of CRC^40^,^41^, the increased frequency of activated/memory MAIT cells in the tumor tissues may participate in anti-tumor immunity against CRC and be associated with the survival of CRC patients. We have followed up those patients for six months after CRC surgical treatment and there was not a single patient ending with death, CRC recurrence or metastasis. We are interested in further following up these patients to investigate the prognostic value of MAIT cells in this population.

We also found that high levels of TNF-α, IFN-γ, IL-2 and IL-17, but not IL-4 and IL-10, were expressed by activated MAIT cells from CRC patients and HC. These data support the notion that activated MAIT cells function like Th1 and Th17 cells^12^,^42^. Interestingly, we detected significantly lower levels of TNF-α and IFN-γ, but higher levels of IL-17 in the supernatants of cultured circulating MAIT cells from CRC patients, as compared with that from the HC following activation *in vitro*. Furthermore, significantly higher levels of IFN-γ and IL-17 mRNA transcripts were observed, and were positively correlated with the levels of TCRVα7.2-Jα33 in the tumor tissues. Together, these data further indicated that higher frequency of activated and memory MAIT cells accumulated in the CRC tissues.

In addition, co-culture of activated MAIT with HCT116 cells significantly reduced the viability of HCT116 cells in a MR1-dependent manner. Recent studies have shown that MAIT cells can be activated by microbial vitamin B metabolites presented by MR1^7^,^8^. Although we did not add any microbial reagents in our cell culture we can not completely rule out the possibility that the culture medium contained unknown MAIT ligands. Currently, there is no information on the exact endogenous MAIT ligands. However, there is considerable evidence supporting that endogenous ligands are present to activate MAIT cells. First, an endogenous ligand is present in the groove of MR1 on thymic hematopoietic cells for the development of MAIT cells^13^. Second, before MR1 is transported to the cell surface, it undergoes a conformational change from an open form to a folded form after binding of a ligand in cytoplasm, which servers similar function of exogenous ligands^43^. Furthermore, all known ligands are presented through interactions with residues lining the A‘ pocket of MR1. Currently, it is unclear whether there are other ligands that are potentially presented in the F‘ pocket of MR1^44^. Therefore, it is possible that some endogenous or exogenous non-B vitamin small molecules may be presented by MR1 on HCT116 cells, which may activate MAIT cells and affect the viability of HCT116 cells in an MR1-dependent manner.

Previous studies have shown that IFN-γ is crucial for T cell immunity against CRC and can regulate the p53 signaling to induce tumor cell cycle arrest and apoptosis^24^,^45^,^46^, while IL-17 expression in the tumor tissues are associated with poor prognosis of patients with early stage of CRC^47^. Collectively, it is possible that during the development and progression of CRC, MAIT cells redistribute and accumulate in the tumor tissues. The MAIT cells from mucosal tissues, such as the lamina propria and from circulation to the tumor tissues may regulate the progression of CRC by directly inducing tumor cell cycle arrest and indirectly regulating tumor and inflammation-related T cell immunity. However, the precise roles of MAIT cells in the development and progression remain further determined.

In summary, our data indicated there were significantly lower frequency of circulating MAIT cells but higher percentages of MAIT cells in the tumor tissues in CRC patients. The percentages of MAIT cells in the tumor tissues were positively correlated with the levels of serum CEA in patients with advanced CRC. Functionally, activated MAIT cells predominantly expressed both IFN-γ and IL-17 and induced human CRC cell cycle arrest in a contact- and MR1-dependent manner *in vitro*. These data indicated that activated and memory MAIT cells accumulated in CRC tumor tissues, regulating the development and progression of CRC. Therefore, our findings may provide new insights in the role of MAIT cells in regulating the progression of CRC.

## Materials and Methods

### Human samples

A total of 48 patients with newly diagnosed CRC were enrolled at the First Hospital of Jilin University, Changchun, China from August 2013 to September 2014. The patients with CRC were diagnosed by histological examination of biopsied tumor tissues obtained from colonoscopy. The levels of serum CEA in individual patients were evaluated. Individual postoperative tumor samples were evaluated, according to the TNM classification system of International Union against Cancer (Edition 7). Five patients with advanced stage of CRC were also recruited in this study, and they had received postoperative chemotherapy with FOLFOX4 (Supplementary Materials and Methods).

Patients with stage I/II of CRC were considered as early stage while those with stage III/IV of CRC were grouped as advanced stage. The CRC patients who had a history of autoimmune and infectious diseases, received preoperative or chemotherapy and immunotherapy were excluded from this study.

In addition, 22 gender- and age-matched healthy volunteers were recruited from the Health Examination Center of the same hospital and their blood samples served as healthy controls (HC). Moreover, the non-tumor tissue (NT) control samples were obtained from another 23 patients with hemorrhoids, when they underwent a procedure for treatment of prolapse and hemorrhoids. These control patients had no other gastrointestinal disease. Their LPMC were isolated.

A total of 42 freshly resected surgical tumor specimens were carefully dissected to separate the surrounding non-tumor tissues from central colorectal cancer tissues. The NIL and TIL were isolated as described previously with minor modification^48^. A portion of NIL and central colorectal cancer samples was used for quantitative real-time PCR analysis, and the remaining samples were used for immunofluorescent assay.

The resected colorectal samples and other specimens were digested with Liberase TM and DNase I (Roche) for isolating LPMC, NIL and TIL. The detailed protocol was described in Supplementary Materials and Methods. Blood samples were collected from HC and CRC patients, and peripheral blood mononuclear cells (PBMC) were obtained by density-gradient centrifugation using Ficoll-paque Plus (GE Healthcare).

This study was approved by the Medical Ethics Committee of the First Hospital of Jilin University and the methods were carried out in accordance with the approved guidelines. Written informed consent was obtained from individual participants in accordance with the Declaration of Helsinki.

### Flow cytometry

The isolated cells were stained with the fluorescent-labeled antibodies, including anti-CD3-PE-CF594 (UCHT1), anti-CD8-V500 (RPA-T8, BD Horizon), anti-CD161-BV421 (DX12), anti-CD4-PerCP-Cy5.5 (RPA-T4), anti-TCRγδ-PE (B1), anti-CD45RO-APC-H7 (UCHL1, BD PharMingen), anti-IL-18Rα-FITC (H44), anti-TCR Vα7.2-APC (3C10, Biolegend) and anti-CCR6-PE (R6H1, eBioscience). The stained cells were characterized on the FACS Aria II flow cytometers (BD Biosciences) and analyzed using the FlowJo software (TreeStar).

### Immunofluorescence

Because of technical limitations in staining surface markers, such as Vα7.2, IL18Rα and CCR6, in formalin-fixed paraffin-embedded tissue sections we stained CD3, CD161, and MDR1 expression to identify MAIT cells in the tissue sections. Our protocol was similar to that in previous reports by co-staining CD3, CD8, and MDR1 to identify MAIT cells in tissue sections^5^,^15^. The colorectal tissue samples were fixed by formalin and paraffin-embedded. The tissue sections (4 μm) were dehydrated and subjected to antigen retrieval in a pressure cooker. After being blocked with donkey sera from healthy animals, the sections were stained with antibodies against CD3 (rabbit polyclonal, Abcam), CD161 (goat polyclonal) or multiple drug resistance-1 (MDR-1) (mouse monoclonal, Santa Cruz Biotech) at 4 °C overnight and the bound antibodies were detected using the corresponding donkey anti–rabbit-Tritc (Abcam), donkey anti–goat-A633 (Molecular Probes), or donkey anti–mouse-A488 (Abcam). The sections were mounted with Prolong Gold with DAPI (Invitrogen) and were photographed under an Olympus FV1000 confocal microscope. The numbers of MAIT cells in three fields (magnification × 200) selected randomly from individual non-tumor and tumor samples were counted in a blinded manner and the data were analyzed with ImageJ (National Institutes of Health).

### MAIT cell activation and cytokine detection

PBMC were stained with anti-CD3-PE-CF594, anti-CD161-BV421, anti-TCRVα7.2-APC to purify MAIT cells by sorting on the FACS Aria II flow cytometer. The purified CD3^+^CD161^+^Vα7.2^+^ MAIT cells were stimulated in duplicate with phorbol 12-myristate 13-acetate (PMA) and ionomycin for 48 h and cultured with brefeldin A (Sigma-Aldrich) for another 4 h (details in the Supplementary Methods). Their supernatants were harvested and the levels of cytokines were analyzed by CBA using the Human Th1/Th2/Th17 Cytometric Bead Array, according to the manufacturer’s instructions (BD Biosciences).

### Quantitative real-time PCR

Total mRNA was extracted from the samples using the Promega RNAeasy Plus Kit (Promega), and reversely transcribed into cDNA with the Superscript II reverse transcriptase (Invitrogen), according to the manufacturer’s instructions. The relative levels of TCR Vα7.2-Jα33, TNF-α, IFN-γ and IL-17A mRNA transcripts to the control GAPDH in individual samples were determined by quantitative real-time PCR using the SYBR Green Mastermix (Invitrogen) and specific primers on a 7500 Fast Real-time PCR system (Applied Biosystems). The primer sequences are present in the Supplementary Methods. The data were analyzed by 2^−ΔΔCt^ method.

### Transwell co-culture system

HCT116 cells were cultured in 10% fetal bovine serum (FBS) RPMI 1640 (Gibco). HCT116 cells (3.0 × 10^5^/well) were transfected with 100 ng GFP-expressing plasmid (pcDNA-GFP) using Lipofectamine 2000 (Invitrogen) per the manufacturers’ instruction.

PBMC were isolated from five HC and MAIT cells were purified by flow cytometry sorting. A preparation of MAIT cells with a purity of >95% was used for the following experiments. To determine the potential function of MAIT cells, some purified MAIT cells were activated with PMA and ionomycin for 6 h. The unstimulated or activated MAIT cells (1.0 × 10^5^/well) were co-cultured in triplicate with the same numbers of GFP^+^ HCT116 cells in the bottom chambers of 24-well transwell plates in 10% FBS RPMI 1640 medium in the presence or absence of 10 μg/ml of anti-MR1 (Abcam) for 48 h as the contact co-culture. In addition, HCT116 cells in the bottom chambers and MAIT cells in the top chambers were non-contact co-cultured in the same condition, as described above for 48 h. The unstimulated, activated MAIT cells or GFP^+^ HCT116 cells in medium alone served as the controls.

Subsequently, the levels of TNF-α, IFN-γ and IL-17A in the supernatants of different groups of co-cultured cells were analyzed by CBA. The GFP^+^ HCT116 cells were analyzed for their cell cycling by flow cytometry. In addition, the unstimulated or activated MAIT were co-cultured with GFP^+^ HCT116 cells at ratios of 10-1:1 (M:H) in either contact or non-contact manner for 48 h and the cell cycle status of HCT116 cells was determined. Moreover, we performed the EdU incorporation assay to determine the impact of MAIT cells co-cultured with HCT116 cells on the viability of HCT116 cells *in vitro* (details seeing the Supplementary Methods).

### Cell cycling analysis

The cell cycling status of HCT116 cells after contact or non-contact co-culture with activated MAIT cells was examined by flow cytometry. The co-cultured cells were harvested and fixed in ethanol overnight at −20 °C. After being washed with PBS three times, the cells were digested with 1 mg/ml of RNaseA and stained with 10 μg/ml of propidium iodide (PI solution). After being washed, the cells were gated on GFP^+^ and the different phases of HCT116 cells were examined on a FACS Calibur flow cytometer using the Cell Quest software (BD Biosciences).

### Statistical analysis

Data are expressed as mean values unless specified. The difference between groups was analyzed by paired or unpaired t tests or Wilcoxon signed-rank test if appropriate. Other data were analyzed by the two-way ANOVA and post hoc Bonferroni tests using the Prism Version 5 (GraphPad). The potential correlation between variables was analyzed by the Spearman rank correlation test. A *P*-value of ≤0.05 was considered statistically significant.

## Additional Information

**How to cite this article**: Ling, L. *et al*. Circulating and tumor-infiltrating mucosal associated invariant T (MAIT) cells in colorectal cancer patients. *Sci. Rep*. **6**, 20358; doi: 10.1038/srep20358 (2016).

## Supplementary Material

Supplementary Information
