# Supplementary material for: Circulating and tumor-infiltrating mucosal associated invariant T (MAIT) cells in colorectal cancer patients
Source: Sci Rep. 2016 Feb 3;6:20358. doi: 10.1038/srep20358 (PMC4738248; doi:10.1038/srep20358)
Supplement: Supplementary Information [file srep20358-s1.pdf]

Circulating and tumor-infiltrating mucosal associated invariant T (MAIT) cells in colorectal cancer patients

Limian Ling[1], Yuyang Lin[1], Wenwen Zheng[1], Sen Hong[1], Xiuqi Tang[1], Pingwei Zhao[1], Ming Li[2], Jingsong Ni[2], Chenguang Li[1], Lei Wang[1*], Yanfang Jiang[2,3*]

Supplementary Methods

Supplementary Figure Legends and Figure

Supplementary Table 1

Supplementary Methods

FOLFOX4 regimen

Individual patients were treated intravenously with Leucovorin (200 mg/m^2) and oxaliplatin (85 mg/m^2) over 2 h and then with 400 mg/m^2 5-FU as a bolus over 10 min, followed by infusion with 600 mg/m^2 5-FU over 22 h on day 1. The patients were treated with 5-FU and leucovorin in the same manner on day 2 for a cycle. The treatments were repeated every 2 weeks for 6 cycles.

Isolation of LPMC, NIL and TIL

The resected colorectal samples were collected into a sterile petri dish containing calcium- and magnesium-free Hank's Balanced Salt Solution (HBSS, Sigma-Aldrich) on ice. The colorectal samples were incubated for 15 min in a shaker at 37˚C in HBSS containing 1 mM DTT, 5 mM EDTA and 0.1% v/v β-mercaptoethanol (Sigma-Aldrich). The remaining tissues were cut into pieces and digested with 125 μg/ml Liberase TM and 250 μg/ml DNase I (Roche) in HBSS at 37˚C for 30 min. The suspending cells were filtered through a 40 μm nylon mesh and lymphocytes were isolated by Ficoll-paque Plus medium (GE Healthcare).

MAIT cell activation

PBMCs were stained with anti-CD3-PE-CF594, anti-CD161-BV421, anti-TCRVα7.2-APC for the purifying MAIT by sorting on the FACS Aria II flow cytometers. The purified CD3+CD161+Vα7.2+ MAIT cells (1.0×105, >95% purity) from individual subjects were stimulated in duplicate with 20 ng/ml phorbol 12-myristate 13-acetate (PMA) and 1 μg/ml ionomycin in 10% human AB sera RPMI 1640 (Gibco) supplemented with 100 U/ml penicillin, 100 μg/ml streptomycin, 50 μg/ml gentamicin, 2 mM L-glutamine and 10 mM HEPES in a

humidified incubator with 5% carbon dioxide for 48 hours and cultured for another 4 hours in the presence of 0.5 μg/ml of brefeldin A (Sigma-Aldrich).

Specific primers

The sequences of specific primers were designed using Primer Express 3.0 (Applied Biosystems) and they were F: GTCGGTCTAAAGGGTACAGT and R: ATTTAGAGTCTCTCAGCTG for TCR Vα7.2-Jα33 (183 bp); F: ACCTCAGGGCTAAGAGCGCA and R: CTGACTGCCTGGGCCAGAGG for TNF-α (143 bp); F: TTTTAATGCAGGTCATTCAGATGT and R: AAGTTTGAAGTAAAAGGAGACAATTTGG for IFN-γ (127 bp); F: CATGAACTCTGTCCCCATCC and R: CCCACGGACACCAGTATCTT for IL-17A (102 bp); F: GCCTCAAGATCATCAGCAAT and R: GCCATCCACAGTCTTCTGGG for GAPDH (143 bp).

EdU incorporation assay

The impact of co-culture of MAIT with HCT116 cells on the proliferation of HCT116 cells was measured by 5-ethynyl-20-deoxyuridine (EdU) incorporation assay using a EdU assay kit (Ribobio, Guangzh China), according to the manufacturers' instructions. After co-culturing for 48 h , the cells in each well were labeled with 200 μM EdU for additional 2 h at 37°C. The cells were fixed with 4% formaldehyde for 30 min at room temperature and permeabilized with 0.5% Triton X-100 for 20 min at room temperature. After being washed with PBS three times, the cells in each well were treated with 200 μl of 1× Apollo® reaction cocktail for 30 min at room temperature. subsequently, the cells were stained with 200 μl of Hoechst33342 for 30 min and visualized under a fluorescent microscope (Olympus Corporation, Tokyo, Japan). The EdU positive cells (green cells) were counted using Image-Pro Plus (IPP) 6.0 software (Media

Cybernetics, Bethesda, MD, USA). The HCT116 cell viability was expressed as the percentages of EdU+ cells in total Hoechst33342+ cells (blue cells). All experiments were done in triplicate and three independent repeating experiments were performed.

Supplementary Figure Legends

(a) The percentages of circulating conventional T cells ($CD4^+$ or $CD8^+$ subsets) in $CD3^+$ T cells in healthy control (HC) (n=22) or CRC patients (n=48). (b) The percentages of $CD45RO^+IL\text{-}18R\alpha^+$ in $CD8^+$ MAIT cells in TIL (n=16), HC (n=13) and NIL (n=16). (c) CCR6 co-stained with MAIT cell antibodies in circulating and tissue from 10 non-tumor control and 10 CRC patients. Both circulating and tissue MAIT cells exhibited high levels expression of CCR6, and the expression of CCR6 has no significantly difference between the two sources of MAIT cells. (d) The numbers of MAIT cells. The mean numbers of MAIT cells in peripheral blood for HC is 39.57; Early CRC is 12.56; Advanced CRC is 5.05 (cells/µl). The numbers of MAIT cells in the tissues; NT: 30.49; for Early CRC, NIL: 25.61 and TIL: 64.46; for Advanced CRC, NIL: 23.56 and TIL :69.84 (cells/µl). (e) The frequencies of circulating MAIT cells in HC (n=22), stage I (n=4), stage II (n=20), stage III (n=19) and stage IV (n=5) CRC patients. (Left) The frequencies of tissue MAIT cells in HC (n=13), stage I (n=2), stage II (n=14), stage III (n=14) and stage IV (n=2) CRC patients. (Right). (f) The FACS gating strategy for MAIT cells sorting ($CD3^+V\alpha7.2^+CD161^+$ MAIT cells were sorteded by flow cytometry.) and their cytokine assessment. (g) EdU incorporation assay to show that co-culture with HCT116 cells significantly reduced the viability of HCT116 cells, which was abrogated by treatment with anti-MR1.

Supplementary Figure

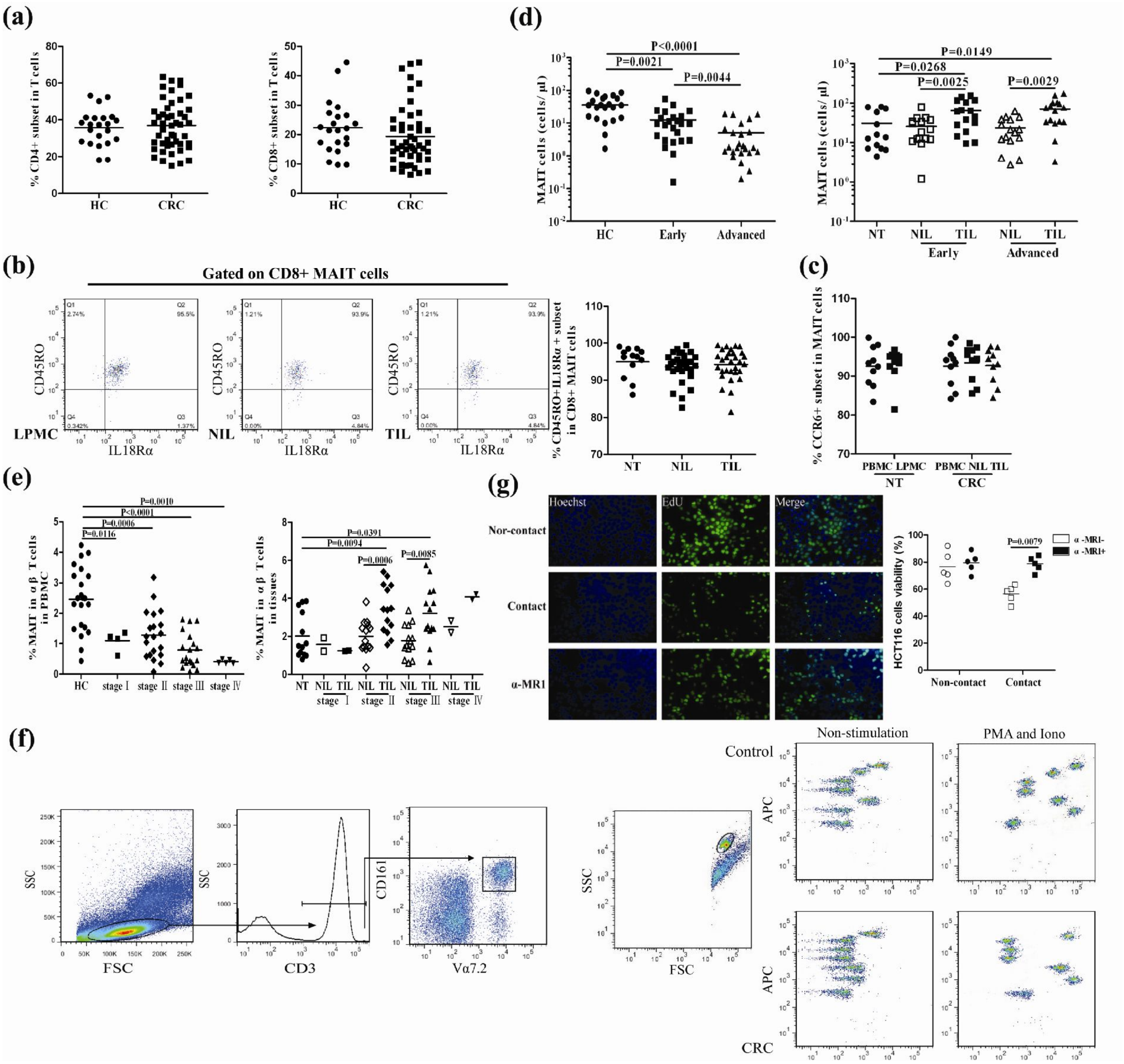

Supplementary Table 1. The baseline demographic and clinical characteristics of subjects

Characteristics	Blood			Tissue		
	**HC (n=22)**	**Early CRC (n=24)**	**Advanced CRC (n=24)**	**NT (n=13)**	**Early CRC (n=16)**	**Advanced CRC (n=16)**
**Age** (year)	62(44-80)	57(46–82)	60 (43-76)	55 (46-72)	57 (46–71)	56 (45-70)
**Sex** (Male/female)	12/10	13/11	10/14	9/4	8/8	10/6
**Body mass index** (kg/m^2)	26 ± 2.7	26 ± 2.2	25 ± 2.3	25 ± 3.0	26 ± 2.4	25 ± 2.5
**Tumor location** (Colon/Rectum)	N/A	7/17	6/18	N/A	6/10	6/10
**TNM stage** (I/II and III/IV)	N/A	4/20 (I/II)	19/5 (III/IV)	N/A	2/14 (I/II)	14/2 (III/IV)
**Differentiation** (good/moderate/poor)	N/A	4/16/4	2/18/4	N/A	2/13/1	3/11/2
**Serum CEA** (ng/ml)	0.52 (0-2.08)	3.61 (0.1-39.2)	21.73 (2.94-98.10)*	0.26 (0-3.17)	4.79(0.63-39.2)	25.15(2.9-82.5)*
**WBC** ($\times10^9$/L)	6.35 (4.85-9.07)	6.29 (4.39-7.10)	7.40 (4.06-10.50)	6.48 (4.51-8.93)	7.19 (5.17-7.10)	7.35 (4.06-10.50)
**Lymphocyte** ($\times10^9$/L)	2.37 (1.27-2.95)	2.13 (1.76-2.78)	2.74 (1.36-3.26)	2.24 (1.27-2.84)	2.23 (1.76-2.78)	2.85 (1.36-3.26)

The normal range of CEA: 0-5 ng/ml; WBC: 4-10 $\times10^9$/L; lymphocytes: 1.2-3.4 $\times10^9$/L. Quantitative variables were reported as mean ± SD and median (range). HC means healthy controls. NT means normal tissue controls. N/A means not applicable. *$p < 0.05$ vs. the controls or early patients.
